# Cardiorespiratory Fitness, Sedentary Behaviour and Physical Activity Are Independently Associated with the Metabolic Syndrome, Results from the SCAPIS Pilot Study

**DOI:** 10.1371/journal.pone.0131586

**Published:** 2015-06-29

**Authors:** Örjan Ekblom, Elin Ekblom-Bak, Annika Rosengren, Mattias Hallsten, Göran Bergström, Mats Börjesson

**Affiliations:** 1 Åstrand Laboratory of Work Physiology, The Swedish School of Sport and Health Sciences, Stockholm, Sweden; 2 Department of Molecular and Clinical Medicine, Institute of Medicine, Sahlgrenska Academy, University of Gothenburg, Gothenburg, Sweden; 3 Sahlgrenska Centre for Cardiovascular and Metabolic Research, Wallenberg Laboratory, Sahlgrenska University Hospital, Gothenburg, Sweden; 4 Karolinska University Hospital, Stockholm, Sweden; Azienda Ospedaliero-Universitaria Careggi, ITALY

## Abstract

**Background:**

Previous studies on the relation between lifestyle and the metabolic syndrome lack one or several aspects of the physical activity pattern in the analyses or cardiorespiratory fitness. Likewise, both uni- and triaxial accelerometry have been used, though, the predictive validity of these two modes has not been compared.

**Objectives:**

The aims of the present study were firstly to investigate the independent relation between cardiorespiratory fitness and physical activity pattern to the metabolic syndrome (MetS) and secondly to examine the predictive validity of uni- and triaxial accelerometry, respectively.

**Methods:**

Data was extracted from the SCAPIS pilot study (n=930, mean age 57.7 yrs). Physical activity pattern was assessed by accelerometry. Cardiorespiratory fitness was estimated using cycle ergometry. MetS was defined per the Adult Treatment Panel III from the National Cholesterol Education Program definition.

**Results:**

Time spent sedentary (OR: 2.38, 95% CI: 1.54-4.24 for T3 vs T1), in light intensity (OR: 0.50, 95% CI: 0.28-0.90) and in moderate-to-vigorous activity (OR: 0.33, 95% CI: 0.18-0.61), as well as cardiorespiratory fitness (OR: 0.24, 95% CI:0.12-0.48), were all independently related to the prevalence of MetS after adjustment for potential confounders, fitness and/or the other aspects of the physical activity pattern. In addition, we found that triaxial analyses were more discriminant, with ORs farther away from the reference group and additional significant ORs.

**Conclusion:**

The finding that several aspects of the physical activity pattern reveal independent relations to the MetS makes new possible targets for behaviour change of interest, focusing on both exercise and everyday life. When assessing the risk status of a patient, it is advised that triaxial accelerometry is used.

## Introduction

The metabolic syndrome (MetS) is an entity, made up of a cluster of cardiovascular risk factors, which increase the risk of future coronary type II diabetes, heart disease, stroke and mortality [1]. The risk for heart failure has been shown to be 2.5-fold in persons with the MetS [2]. Similar increases in risk have been shown for cardiovascular disease, all-cause and cardiovascular mortality and stroke [3]. The prevalence of MetS in Europe is approximately 24% (using the NCEP ATP-III definition), in the middle-aged population [4]. Prevalence varies between countries, partly dependent on the fact that different definitions exist [5]. Lifestyle has been closely associated with the development of MetS, with diet and physical activity (PA) identified as two of the most important modifiable lifestyle factors, in this regard [6].

The PA pattern of an individual can be described as time (or per cent of the awake time) spent pursuing activities of differing intensities, ranging from sedentary behaviours (SED, defined as PA intensities below 1.5 METs) and light intensity PA (LIPA, intensities between 1.5 and 3 metabolic equivalents, METs) to PA of moderate- and vigorous intensity (MVPA, above 3 METs). Other aspects of the PA pattern includes even more detailed measures, such as the presence of prolonged, uninterrupted periods (“bouts”) spent in (less beneficial) SED or in (more beneficial MVPA), as well as the frequency by which the prolonged periods of SED is interrupted. Different aspects of the PA pattern have previously been associated with increased risk of MetS development [7–11]. In a recent meta-analysis on longitudinal studies using self-reported PA, Huang and co-workers found lower incidence of MetS in subjects with moderate or high levels of leisure-time PA compared to low levels [12]. Moreover, in a meta-analysis including cross-sectional studies, Edwardson and co-workers reported 73% higher prevalence of the MetS in subjects with more sedentary time, compared to those with the least sedentary time [7]. Interestingly, no effect was found in a sensitivity analysis where the effect of sedentary behaviour was studied in different PA strata, indicating an important effect of sedentary behaviour in it self. All studies but one were based on self-reports. However, studies using self-reports and focusing only on leisure-time PA may miss much of the inter-individual variation in the PA pattern. Using self-report methods to quantify SED or MVPA, have previously been shown to underestimate the strength of the association to cardiovascular risk factors [13], compared to objectively accelerometer data. In addition to PA pattern, cardiovascular fitness (expressed as maximal aerobic capacity, VO_2_max) has also been associated with the MetS and other cardiovascular diseases, independently of adiposity and PA patterns [11,14,15].

Several studies have included several, albeit not all, aspects of the physical activity pattern and metabolic risk, in both adults [16] and in children [17]. Only one previous study has assessed the risk of MetS with respect to cardiorespiratory fitness, SED and PA simultaneously. The result from a study by Shuval and co-workers based on the Cooper Center Longitudinal Study (CCLS) [14] stresses the modulating effect of cardiorespiratory fitness on the relation between PA patterns and MetS. However, the analyses were based on a sub-sample of the CCLS, and used self-reported data, in which sedentary time is especially approximate. Detailed information on the relation between objectively assessed PA patterns, cardiorespiratory fitness and the MetS is therefore still needed, not least to form the basis for future PA-recommendations in the clinical setting.

Since the technologies and analysis manners for assessment of PA patterns are evolving, it is of importance to study predictive validity of the different, new techniques available. Most of the recently published research regarding the relation between PA, SED and health outcomes is based on uniaxial accelerometry. However, almost all contemporary monitors are triaxial, and are thereby potentially able to capture more or other aspects of the actual movements performed. However, to what extent three-dimensional accelerometry have a stronger predictive validity for health outcomes such as the MetS, is not yet known.

Thus, the aims of the present study, were firstly to investigate the independent relation between cardiorespiratory fitness, SED and PA to the MetS and secondly to examine the predictive validity of uniaxial and triaxial accelerometry, respectively, for the presence of MetS.

## Materials and Methods

Accelerometer data was derived from the Swedish CArdioPulmonary bioImage Study (SCAPIS) pilot study, conducted at the Sahlgrenska University Hospital in Göteborg, Sweden. In 2012, a randomly selected population sample including 2243 adults aged 50 to 65 years were recruited from the census register, stratified for low and high socioeconomic status area. Out of these, 1111 (49.5%) agreed to participate. During two days, the participants underwent extensive imaging and functional studies of the heart, lungs and metabolism. They also completed an extensive questionnaire regarding i.e. lifestyle and living conditions, as well as performed a submaximal cycle test to determine cardiorespiratory fitness. Further, subjects wore an accelerometer during seven days, to objectively register the daily PA pattern. The study was approved by the Umea ethical board (Dnr 2010-228-31M), and all participants provided written informed consent.

### Measurement of sedentary behaviour and physical activity

ActiGraph accelerometers (model GT3X and GT3X+, ActiGraph LCC, Pensacola, FL, USA) were used to objectively measure the daily movement pattern. The accelerometer was handed to the participant during the second day of the visits to the test centre. The participants were instructed to wear the accelerometer in an elastic belt over the right hip during all waking hours for at least 7 consecutive days, except during water based activities. After the registration period, the accelerometer was returned by prepaid mail. The accelerometer was initialized and downloaded using the ActiLife v.6.10.1 software. Raw data sampling frequency was set to 30 Hz, and extracted as 60-s epoch with low frequency extension filter for the present analyses. Both uniaxial and triaxial analyses were performed to enable comparisons with previous research and to compare predictive validity.

### Accelerometer data processing

A total of 1067 participants (96.0%) agreed to wear an accelerometer. Minimum requirement for data inclusion was 600 minutes of valid daily monitor wear on at least 4 days. Wear time was defined by subtracting non-wear time from 24 h. Non-wear time was defined as at least 60 consecutive minutes with no movement (0 counts per minute, cpm), with allowance for maximum 2 minutes of cpm between 0 and 100 (uniaxial) and 0 and 200 (triaxial). In total, seven accelerometers were lost by the participants or in mail transport and another 112 measurements provided invalid data (due to insufficient wear time, labelling error or accelerometer malfunction). The majority of the included participants had valid data for at least 7 days (67%), 19% for 6 days, 9% for 5 days, and 5% for 4 days. A total of 948 participants provided valid uniaxial accelerometer data A total of 18 subjects did not provide sufficient data on subcomponents of MetS and 930 participants were included in the uniaxial analyses. Another six participants could be included in the tri-axial analyses, since their accelerometer data contained sufficient amount of data. Subject characteristics are based on subjects with sufficient uniaxial data and data on MetS.

The daily PA pattern is presented as 1) percentage of wear time spent in three intensity-specific categories; sedentary [SED], light [LIPA] and moderate-to vigorous [MVPA] PA, 2) total volume of PA expressed as mean cpm over the study period [TPA], 3) time spent in prolonged periods of SED (bouts of ≥20 consecutive minutes below SED threshold, with no allowance for interruption above threshold) [SED bouts], 4) number of breaks per sedentary hour (interruption in SED from one minute below threshold, to the following minute above threshold) [SED breaks], and 5) fulfilment of national PA recommendations (see below).

### Uniaxial accelerometer analysis

In order to identify intensity-specific categories, absence of or very low registrations (<100 cpm) was accepted as SED, according to standards [18], while cpm between 100 and 2019 were defined as LIPA, and cpm ≥2020 as MVPA with no further distinction between moderate- and vigorous PA [19,20]. Mean cpm to express TPA for the uniaxial analysis, was defined as the total numbers of counts divided by minutes of wear time.

### Triaxial accelerometer analysis

As an additional way of analysing accelerometer data, the sampled acceleration from all three axes in the 60-s data was combined into the vector magnitude (VM). In these analyses, time was regarded as SED when spent in intensities between 0 and 199 cpm [21]. LIPA was regarded as cpm between 200 and 2689, and MVPA as ≥ 2690 cpm, using standard definitions [22]. Mean cpm to express TPA for the triaxial analysis, was calculated as total VM divided by minutes of wear time.

### National recommendations

Current Swedish national guidelines recommend at least 150 minutes per week of MVPA (above three times the resting metabolic rate, equivalent to a brisk walk for many individuals) preferably on most days of the weeks, in bouts of 10 minutes or more (www.yfa.se). We chose to evaluate the importance of fulfilling these criteria of 150 minutes of MVPA per week with regard to MetS prevalence, both in strict line with the recommendations (30 minutes per day on at least 5 of 7 days of the week, of which all are from prolonged bouts of 10 minutes or more) but also using a less strict interpretation of said recommendations, without the requirement of regularity and accumulation in prolonged bouts (accumulating a total of 150 MVPA minutes per week).

### Fitness testing

All subjects were invited to perform a submaximal aerobic fitness test. A total of 130 subjects (14.1%), did not participate, mainly due to pain (knee, lower back or hip), obesity, perceived inability to perform a test, on-going illnesses or due to malfunction of heart rate monitors or ergometer. Participants with a diagnosed heart condition or taking beta-adrenergic blockers were excluded a priori. This group represented the majority of excluded participants. Estimated cardiorespiratory fitness was estimated from the difference in heart rate response to two submaximal work rates in relation to the difference in work rate. The test was performed on a Monark ergometer 839E (Monark Exercise AB, Vansbro, Sweden) at 60 rpm. Heart rate was measured using telemetry (Polar Electro Oy, Tampere, Finland). Data was then normalised for body mass and expressed as mL·min^-1^·kg^-1^. The criterion validity of the method has been tested [23], using direct measurement as criterion method (r^2^ = 0.91 [95% CI: 0.87 to 0.93], SEE: 0.302 L per min).

### The metabolic syndrome

Participants were classified as either having or not having the metabolic syndrome, according to the National Cholesterol Education Program (NCEP), 2001 definitions [1]. The NCEP Adult treatment Panel III (ATPIII panel), defined the metabolic syndrome as the presence of three or more of the following: fasting plasma glucose ≥ 6.1 mmol·l^-1^, serum triglycerides ≥ 1.69 mmol·l^-1^, serum HDL-cholesterol ≤ 1.04 mmol·l^-1^, in men and ≤ 1.29 mmol·l^-1^ in women, blood pressure ≥ 130/85 mmHg, or waist circumference ≥ 102 cm in men and ≥ 88 cm in women.

### Other measurements

Measurements of weight, height and waist circumference were performed during the first visit to the test centre. Age was categorized into 3 levels (50 to 54, 55 to 59, and 60 to 65 years). Through self-administrated questionnaire responses, education level was dichotomized into university degree or not, smoking habits were dichotomized into regular smoker or not, and perceived psychosocial stress divided into four levels. An extensive food frequency questionnaire [24] was used to assess food habits, and answers were used to calculate daily caloric intake [EI]. Data for EI were categorized into quartiles (Q1: ≤ 1332, Q2: 1333–1751, Q3: 1752–2294 and Q4: ≥ 2295 kcal·d^-1^).

### Statistical analysis

Data was checked for normality using the Shapiro-Wilk test. The majority of the variables were skewed, and hence the descriptive data is presented as proportions or median and 25^th^-75^th^ percentile (Q1-Q3). Fitness and accelerometer data were arbitrarily divided into tertiles. Fitness tertiles were computed gender-specific and tertiles of physical activity behaviour (accelerometer data) were calculated with genders merged. Those not participating in the fitness testing did not differ from completers regarding gender, perceived psycho-social stress or energy intake (chi-square all p>0.2), but were more likely to be smokers, have low education level and MetS (chi-square all p<0.05). Cut-offs are given in Table 1.

**Table 1:** Cut-off-values for fitness and accelerometer data (uniaxial and triaxial data, respectively).

Accelerometer analyse mode	Variable	Cut-off Tertile 1 –Tertile 2	Cut-off Tertile 2 –Tertile 3
	Fitness (ml·min^-1^·kg^-1^)	W: 26.3, M: 36.8	W: 32.1, M: 41.1
**Uniaxial**	SED (% of wear time)	57%	64%
	LIPA (% of wear time)	32%	39%
	MVPA (% of wear time)	3%	5%
	TPA (cpm)	289	390
	SED_bouts (min per day)	153	228
	SED_breaks (no per day)	9.1	11.2
**Triaxial**	SED (% of wear time)	49%	58%
	LIPA (% of wear time)	37%	45%
	MVPA (% of wear time)	4%	7%
	TPA (cpm)	550	721
	SED_bouts (min per day)	123	200
	SED_breaks (no per day)	9.0	11.5

Odds ratios (ORs) with 95% confidence interval (95% CI) were calculated using binominal logistic regression, controlling for a) age and gender, b) age, gender, educational level, smoking status, psychological stress and caloric intake, c) SED, LIPA and MVPA when not evaluated and d) fitness. When LIPA or SED were used as independent variables, the other of the two was excluded to avoid multi-collinearity (as rho = 0.89 and 0.96 for uniaxial and triaxial data, respectively). When the 95% CI did not include the reference value of 1, ORs were considered as significantly elevated/lowered. Adding wear time (either average minutes per day or number of days) as an independent variable altered the results only in a minimal manner. Therefore, to minimize the numbers of independent variables, wear time was taken out from the analyses. In analyses of sub-components of MetS, OR of having elevated values were considered separately for each sub-component. Cut-offs were identical to NCEP 2001 definitions [1]. All statistical analyses were performed by using SPSS (Statistical Package for the Social Sciences for Windows, 14.0, 2006, SPSS Inc., Chicago IL).

## Results

Table 2 shows the subject characteristics using the uniaxial data. Similar results regarding the differences between participants with and without MetS were derived using triaxial data (data not shown).

**Table 2:** Subject characteristics in relation to MetS prevalence or not.

	Metabolic syndrome		
	*No (n = 726)*	*Yes (n = 204)*	Difference p-value**
**Per cent women**	52.1%	52.5%	n.s.
**Median age (yrs.)**	52.7 (53.6–61.4)	58.6 (54.7–62.5)	0.005
**University degree level(%)**	41.3	27.6	0.001
**Regular smokers (%)**	11.6	13.9	n.s.
**High stress (%)** *	20.3	24.1	n.s.
**SED (% of wear time)**	60.2 (54.4–65.8)	62.5 (55.5–68.8)	0.001
**LIPA (% of wear time)**	35.4 (30.4–41.1)	34.3 (28.3–40.9)	0.09
**MVPA (% of wear time)**	4.1 (2.7–5.8)	2.8 (1.7–4.7)	<0.001
**TPA (cpm)**	345 (277–433)	288 (227–376)	<0.001
**Est. VO**_**2**_ **max (ml x min** ^**-1**^ **x kg** ^**-1**^ **)**	35.5 (28.0–40.4)	32.3 (27.4–37.3)	0.002

Data are presented as median (Q1-Q3) or as per cent, where applicable. Differences between MetS-strata were analysed using Mann-Whitney U-test or chi-square test.

*Reporting tension, anxiousness, nervousness or sleep disturbances more or less constantly over the last year or longer

** Chi-square test for nominal data and Mann-Whitney test for ordinal data.

### PA patterns and fitness in relation to MetS, using uniaxial accelerometry

The OR for having the MetS was calculated across fitness, SED and PA tertiles (Table 3). Regarding fitness, participants in the third tertile showed a markedly lower OR compared to the lowest tertile. ORs changed only slightly when taking lifestyle variables and PA pattern variables in consideration. Similarly, participants in the highest tertile of SED had a significant higher OR for MetS, even with both MVPA and fitness accounted for. MVPA was related to lower OR for MetS in a dose-response manner, regardless of SED and fitness. A somewhat weaker relation was found for TPA. Neither LIPA, time in neither SED bouts nor number of SED breaks was significantly associated with increased risk of the MetS after full adjustment.

**Table 3:** Odds ratios (95% CI) for having the MetS in relation to tertiles of fitness and objectively measured PA patterns using uniaxial accelerometry.

			Tertile 2	Tertile 3
**Fitness**		n		
	Age-gender	686	0.70 (0.45–1.10)	**0.26 (0.15–0.47)**
	+Lifestyle	623	0.68 (0.42–1.11)	**0.22 (0.11–0.44)**
	+SED	623	0.69 (0.42–1.13)	**0.24 (0.12–0.47)**
	+MVPA	623	0.77 (0.46–1.26)	**0.26 (0.13–0.52)**
	+SED and MVPA	623	0.76 (0.46–1.26)	**0.27 (0.13–0.54)**
**Accelerometry**				
**SED**	Age-gender	930	0.99 (0.66–1.49)	**1.70 (1.15–2.50)**
	+Lifestyle	831	1.13 (0.71–1.77)	**2.17 (1.41–3.34)**
	+MVPA	831	0.97 (0.61–1.55)	**1.65 (1.05–2.59)**
	+Fitness	623	1.21 (0.66–2.22)	**2.40 (1.34–4.30)**
	+MVPA and Fitness	623	1.08 (0.58–2.02)	**1.87 (1.02–3.43)**
**LIPA**	Age-gender	930	0.70 (0.48–1.04)	0.87 (0.59–1.27)
	+Lifestyle	831	**0.56 (0.37–0.85)**	0.72 (0.47–1.09)
	+MVPA	831	**0.60 (0.39–0.92)**	0.78 (0.51–1.19)
	+Fitness	623	0.62 (0.36–1.07)	0.74 (0.42–1.30)
	+MVPA and Fitness	623	0.65 (0.37–1.13)	0.75 (0.42–1.33)
**MVPA**	Age-gender	930	**0.44 (0.30–0.65)**	**0.33 (0.22–0.49)**
	+Lifestyle	831	**0.44 (0.29–0.67)**	**0.32 (0.20–0.50)**
	+SED	831	**0.47 (0.31–0.71)**	**0.37 (0.23–0.58)**
	+Fitness	623	**0.39 (0.23–0.67)**	**0.27 (0.15–0.49)**
	+SED and Fitness	623	**0.41 (0.24–0.70)**	**0.31 (0.17–0.56)**
**TPA**	Age-gender	930	**0.42(0.29–0.61)**	**0.34 (0.23–0.50)**
	+Lifestyle	831	**0.36 (0.24–0.55)**	**0.33 (0.21–0.51)**
	+SED	831	**0.39 (0.25–0.61)**	**0.37 (0.22–0.62)**
	+MVPA	831	**0.48 (0.29–0.77)**	0.60 (0.29–1.20)
	+Fitness	623	**0.38 (0.22–0.66)**	**0.30 (0.17–0.54)**
	+SED, MVPA and Fitness	623	0.76 (0.37–1.55)	1.18 (0.39–3.63)
**SED bouts**	Age-gender	930	0.88 (0.59–1.32)	1.40 (0.96–2.05)
	+Lifestyle	831	0.97 (0.62–1.51)	**1.85 (1.22–2.82)**
	+MVPA	831	0.89 (0.57–1.41)	1.53 (0.99–2.36)
	+Fitness	623	0.77 (0.43–1.31)	1.68 (0.97–2.90)
	+MVPA and Fitness	623	0.75 (0.41–1.36)	1.42 (0.81–2.50)
**SED breaks**	Age-gender	930	0.79 (0.54–1.16)	0.74 (0.50–1.10)
	+Lifestyle	831	**0.64 (0.52–0.96)**	**0.56 (0.37–0.86)**
	+MVPA	831	0.70 (0.46–1.07)	**0.63 (0.41–0.98)**
	+Fitness	623	0.59 (0.34–1.01)	**0.56 (0.32–0.99)**
	+MVPA and Fitness	630	0.66 (0.38–1.14)	0.59 (0.33–1.14)

Reference categories are participants in the lowest tertile. 95% CI s not including 1 are marked in bold.

Age-gender: Adjusted for gender and age (yrs.)

+ Lifestyle: additionally adjusted for education level (university degree vs. not), energy intake (kcal·d^-1^ in quartiles), smoking habits (regular smoker vs. not) and psychosocial stress (self-reported into four levels)

+ SED: additionally adjusted for % of wear time spent in SED (in tertiles)

+ MVPA: additionally adjusted for % of wear time spent in MVPA (in tertiles)

+ Fitness: additionally adjusted for estimated VO_2_max (ml·min^-1^·kg^-1^, in tertiles)

### PA patterns and fitness in relation to MetS, using triaxial accelerometry

In the second set of analyses, the same analyses as shown in Table 3 were performed, using triaxial accelerometry (Table 4). The main differences in the results between the two sets of analyses was found in the lower part of the intensity spectrum. Using triaxial analyses, not only SED but also higher tertiles of LIPA was significantly associated with the prevalence of the MetS. Furthermore, participants in the highest tertile of time in SED bouts had an increased OR for MetS even after taking MVPA and fitness as separate confounders into consideration. Also, compared to uniaxial analyses, participants in the highest fitness tertile had an equally low OR for MetS, compared to those in the reference group with the lowest fitness values. The associations of MVPA and TPA were comparable between uni- and triaxial accelerometry analyses.

**Table 4:** Odds ratios (95% CI) for having the MetS in relation to tertiles of fitness and objectively measured SED and PA patterns using triaxial accelerometry.

			Tertile 2	Tertile 3
**Fitness**		n		
	Age-gender	782	**0.62 (0.41–0.93)**	**0.22 (0.13–0.37)**
	+Lifestyle	709	**0.59 (0.38–0.92)**	**0.19 (0.10–0.35)**
	+SED	632	0.73 (0.45–1.19)	**0.22 (0.11–0.44)**
	+MVPA	632	0.73 (0.45–1.21)	**0.24 (0.12–0.47)**
	+SED and MVPA	623	0.76 (0.46–1.25)	**0.24 (0.12–0.48)**
**Accelerometry**				
**SED**	Age-gender	937	1.05 (0.62–1.58)	**1.94 (1.31–2.87)**
	+Lifestyle	836	1.08 (0.68–1.71)	**2.39 (1.55–3.67)**
	+MVPA	836	0.93 (0.58–1.49)	**1.72 (1.08–2.74)**
	+Fitness	632	1.30 (0.71–2.38)	**2.38 (1.54–4.24)**
	+MVPA and Fitness	623	1.10 (0.59–2.06)	1.66 (0.90–3.08)
**LIPA**	Age-gender	937	**0.58 (0.39–0.84)**	**0.56 (0.38–0.82)**
	+Lifestyle	836	**0.48 (0.32–0.73)**	**0.43 (0.28–0.67)**
	+MVPA	836	**0.54 (0.36–0.83)**	**0.50 (0.32–0.77)**
	+Fitness	623	**0.57 (0.33–0.97)**	**0.45 (0.25–0.79**)
	+MVPA and Fitness	623	0.65 (0.38–1.13)	**0.50 (0.28–0.90)**
**MVPA**	Age-gender	937	**0.52 (0.36–0.75)**	**0.37 (0.25–0.55)**
	+Lifestyle	836	**0.50 (0.33–0.75)**	**0.34 (0.22–0.53)**
	+SED	836	**0.56 (0.37–0.85)**	**0.42 (0.26–0.68)**
	+Fitness	623	**0.45 (0.26–0.76)**	**0.28 (0.16–0.51)**
	+SED and Fitness	623	**0.48 (0.28–0.83)**	**0.33 (0.18–0.61)**
**TPA**	Age-gender	937	**0.54 (0.37–0.78)**	**0.40 (0.27–0.59)**
	+Lifestyle	836	**0.53 (0.36–0.80)**	**0.32 (0.20–0.50)**
	+SED	836	**0.58 (0.36–0.93)**	**0.37 (0.20–0.69)**
	+MVPA	836	0.66 (0.41–1.04)	**0.48 (0.25–0.93)**
	+Fitness	623	**0.50 (0.30–0.85)**	**0.28 (0.15–0.52)**
	+SED, MVPA and Fitness	623	0.85 (0.40–1.79)	0.75 (0.23–2.42)
**SED bouts**	Age-gender	937	1.20 (0.80–1.79)	**1.81 (1.23–2.67)**
	+Lifestyle	836	1.31 (0.84–2.04)	**2.16 (1.41–3.33)**
	+MVPA	836	1.21 (0.77–1.90)	**1.68 (1.07–2.64)**
	+Fitness	623	1.42 (0.79–2.54)	**2.37 (1.33–4.23)**
	+MVPA and Fitness	623	1.32 (0.73–2.39)	1.79 (0.98–3.27)
**SED breaks**	Age-gender	937	0.79 (0.54–1.14)	**0.61 (0.41–0.91)**
	+Lifestyle	836	0.72 (0.48–1.08)	**0.55 (0.38–0.84)**
	+MVPA	836	0.83 (0.55–1.26)	**0.64 (0.42–1.00)**
	+Fitness	623	0.84 (0.49–1.42)	**0.55 (0.31–0.97)**
	+MVPA and Fitness	623	1.05(0.61–1.83)	0.66 (0.39–1.20)

Reference categories are participants in the lowest tertile. 95% CIs not including 1 are marked in bold.

Age-gender: Adjusted for gender and age (yrs.)

+ Lifestyle: additionally adjusted for education level (university degree vs. not), energy intake (kcal·d^-1^ in quartiles), smoking habits (regular smoker vs. not) and psychosocial stress (self-reported into four levels)

+ SED: additionally adjusted for % of wear time spent in SED (in tertiles)

+ MVPA: additionally adjusted for % of wear time spent in MVPA (in tertiles)

+ Fitness: additionally adjusted for estimated VO_2_max (ml·min^-1^·kg^-1^, in tertiles)

### MetS prevalence in relation to PA recommendations fulfilment

After controlling for age, gender, lifestyle, fitness and SED, meeting the current national PA-recommendations (assessed via uniaxial accelerometry) with requirement of regularity and accumulation of MVPA in prolonged bouts, totalling 150 minutes/week lowered the risk non-significantly (OR = 0.38, 95% CI 0.11 to 1.28). Using the less strict interpretation of the recommendations was associated with a lower OR of having the MetS (OR = 0.30, 95% CI:0.19–0.50).

### Association of the PA pattern with having the subcomponents of the MetS

**Fig 1:**
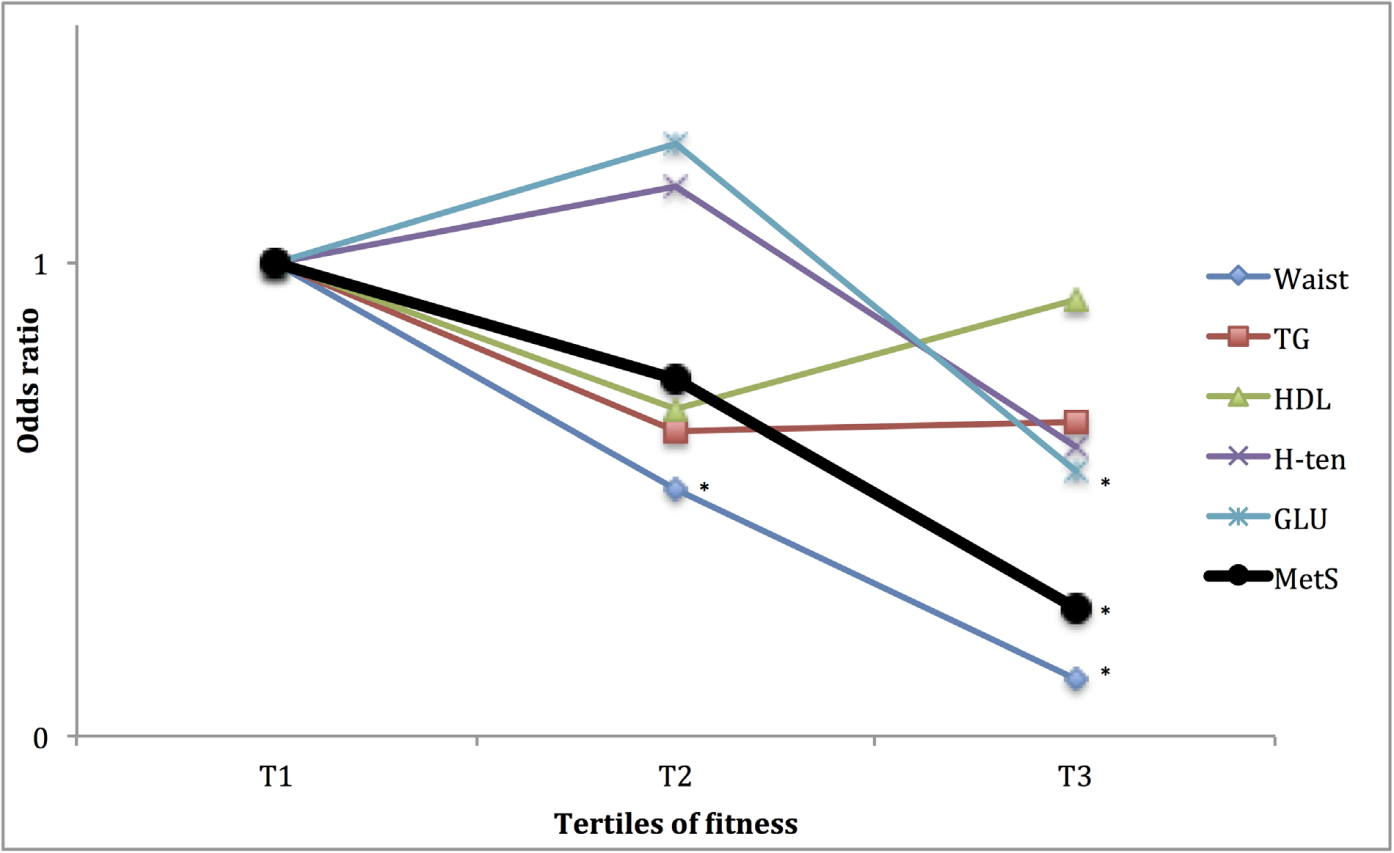
Relationship between tertiles of fitness and having unfavourable levels of sub-components of METs. ORs are adjusted for gender, age, education level (university degree vs. not), energy intake (kcal·d^-1^ in quartiles), smoking habits (regular smoker vs. not) and psycho-social stress (self-reported into four levels), % of wear time spent in SED and % of wear time spent in MVPA (in tertiles). * Denotes significant difference to reference group.

**Fig 2:**
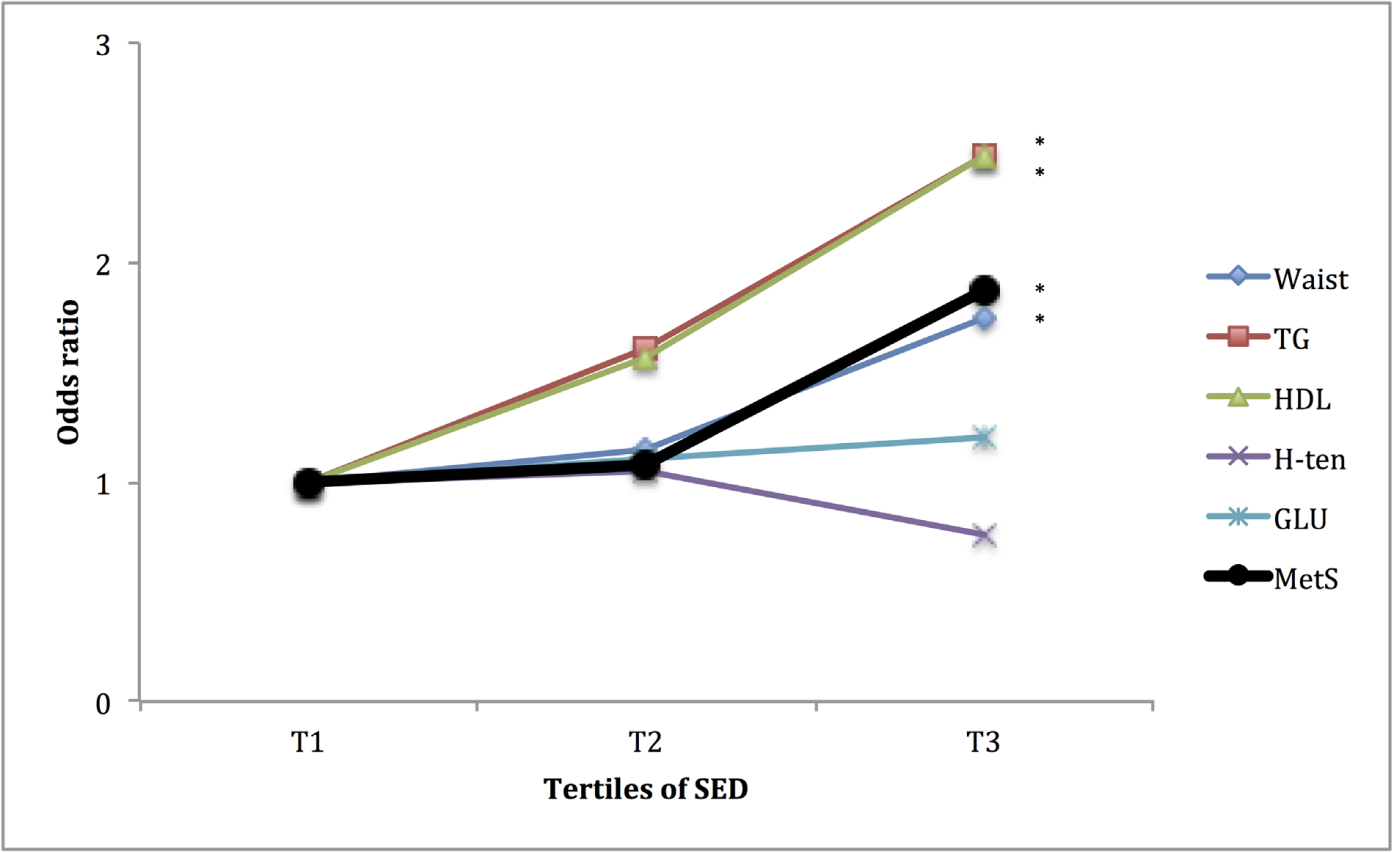
Relationship between tertiles of SED and unfavourable sub-components of METs. ORs are adjusted for gender, age, education level (university degree vs. not), energy intake (kcal·d^-1^ in quartiles), smoking habits (regular smoker vs. not) and psycho-social stress (self-reported into four levels), % of wear time spent in MVPA (in tertiles), and estimated VO_2_max (ml·min^-1^·kg^-1^, in tertiles). * Denotes significant difference to reference group.

**Fig 3:**
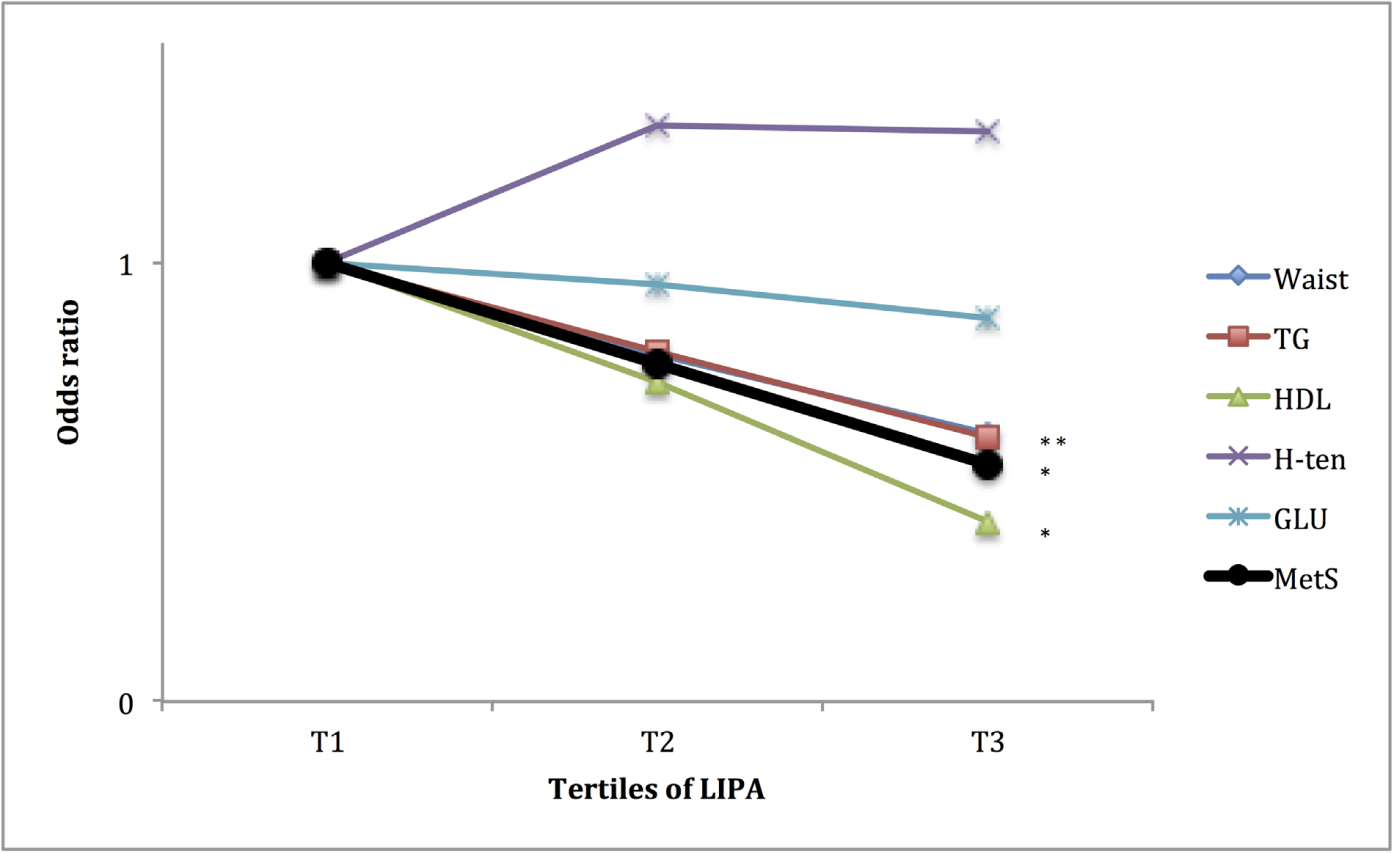
Relationship between tertiles of LIPA and unfavourable sub-components of METs. ORs are adjusted for gender, age, education level (university degree vs. not), energy intake (kcal·d^-1^ in quartiles), smoking habits (regular smoker vs. not) and psycho-social stress (self-reported into four levels), % of wear time spent in MVPA (in tertiles), and estimated VO_2_max (ml·min^-1^·kg^-1^, in tertiles). * Denotes significant difference to reference group.

**Fig 4:**
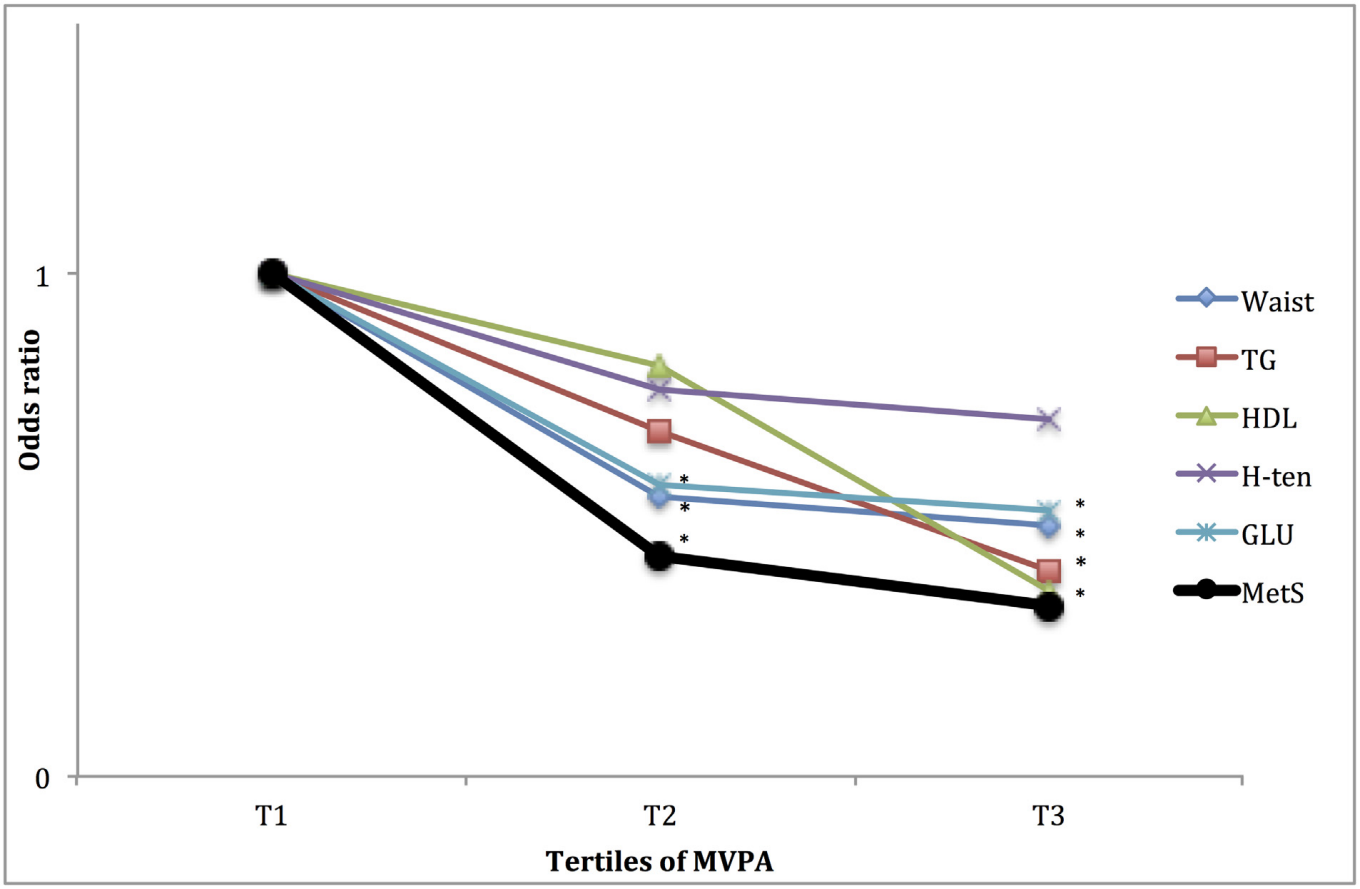
Relationship between tertiles of MVPA and for unfavourable sub-components of METs. ORs are adjusted for gender, age, education level (university degree vs. not), energy intake (kcal·d^-1^ in quartiles), smoking habits (regular smoker vs. not) and psycho-social stress (self-reported into four levels), % of wear time spent in SED (in tertiles), and estimated VO_2_max (ml·min^-1^·kg^-1^, in tertiles). * Denotes significant difference to reference group.

Furthermore, as the MetS is a cluster of unfavourable metabolic and haemostatic conditions, it may be of interest to study how these are individually associated to fitness and PA pattern. In Figs 1 to 4, the associations of fitness and tri-axial SED, LIPA, and MVPA with unfavourable levels of each sub-component, according to the definition of the ATPIII-panel (above) of the MetS are presented. ORs are adjusted for potential confounders as well as, when not evaluated, the other main exposures. Higher fitness tertiles did primarily affect the OR of having unfavourable waist circumference and fasting glucose levels. SED did primarily affect the OR of having unfavourable waist circumference, HDL-cholesterol and triglyceride (TG) levels, with significantly higher OR (SED) and lower OR (LIPA), in the highest tertile, compared to the lowest. MVPA did primarily affect the OR of having unfavourable waist circumference, fasting glucose, HDL-cholesterol and triglycerides levels, with significantly lower OR in tertile 3 compared to tertile 1. ORs for waist circumference and fasting glucose were also lower in tertile 2, compared to tertile 1.

### The association to SED, with regards to MVPA

**Fig 5:**
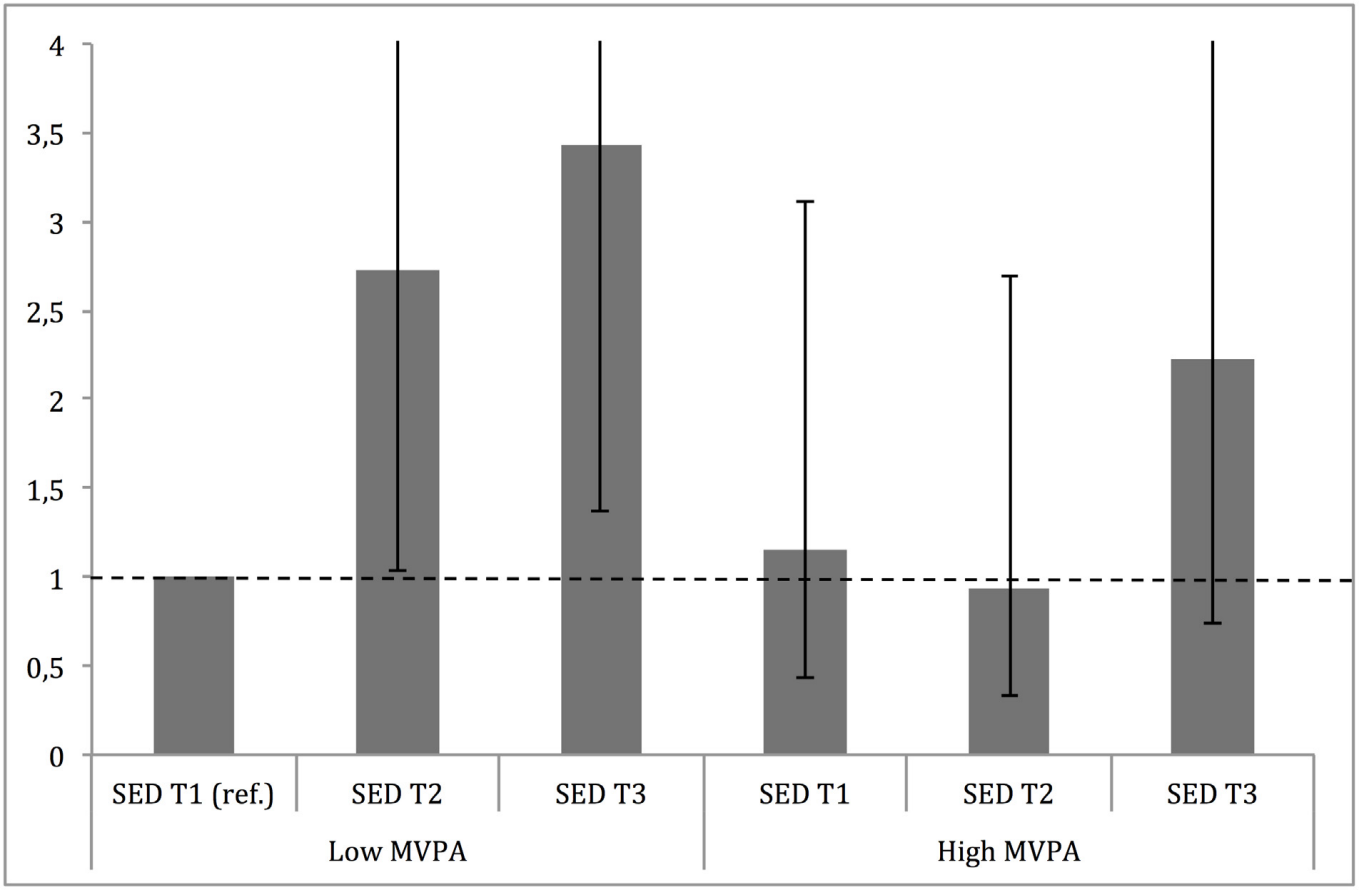
Stratified analysis across SED tertiles in high (above median) and low (below median) MVPA. ORs are adjusted for gender, age (yrs.), education level (university degree vs. not), energy intake (kcal·d^-1^, in quartiles), smoking habits (regular vs. not), psychosocial stress (self-reported into four levels) and fitness (in tertiles).

Finally, the risk for MetS associated with higher SED was analysed with regard to MVPA, stratified into high (above median) and low (below median) MVPA. Results are shown in Fig 5 and are similar, but not identical, to those obtained in the regression analyses. Participants with low MVPA had gradually increased OR for MetS prevalence with increased SED tertiles. In the high MVPA strata, participants with low or moderate SED (T1 and T2) had similar OR as reference group, while participants in the highest SED tertile had a 2-fold increase in OR, albeit not significantly different from the reference group.

## Discussion

The main result of the present cross-sectional study, was that in a sample of middle-aged women and men, several aspects of the daily PA pattern (SED, LIPA and MVPA), and cardiorespiratory fitness, were related to the prevalence of MetS. These results remained significant after controlling for the other exposures, as well as for multiple lifestyle factors.

In addition, we found that uniaxial analyses revealed ORs closer to 1 and fewer significant findings, when compared to triaxial analyses. We therefore argue that triaxial accelerometry was more discriminant for the risk for the MetS, compared to uniaxial accelerometry. This difference in predictive validity seems to be most pronounced in the lower part of the PA spectrum.

Importantly, cardiorespiratory fitness was found to have the strongest association to MetS. The strongest association in the fully adjusted analyses was found for the highest tertile of fitness compared to the lowest, with a reduced OR for the MetS by 73%. However, significant relations were also found for the highest tertile of triaxial captured SED (increased OR by 82%), LIPA (reduced OR by 46%) and MVPA (reduced OR by 56% and 66% for second and third tertile, respectively). This implies the independent importance of cardiorespiratory fitness as well as all aspects of the daily PA pattern for MetS. In this regard, the present findings are in line with previous studies [7–12,15,25]. Even so, the results of the present study also expand on current knowledge, as in previous studies, one or more of the exposures have not been accounted for in the analyses. Only in the study by Shuval and co-workers [14] that mainly focused on the association between sedentary behaviour and cardio-metabolic risk, all main exposures were simultaneously evaluated [14]. They found several significant cross-sectional associations between weekly, self-reported sitting time and cardio-metabolic risk factors after adjusting for PA, but that most associations were blunted when taking fitness into account. In their prospective analyses, increased sitting time at baseline did not affect the MetS incidence during a mean follow-up of 9.3 years when adjusting for PA, fitness and other covariates. This is in contrast to our findings, showing an independent relation for SED, LIPA and MVPA to the MetS, even after adjusting for fitness. In addition, TPA and prolonged bouts of SED were significantly associated with MetS prevalence when the other exposures were separately accounted for, but not in the joint analysis including all. In fact, it is not surprising that these associations did not persist when controlling for all three PA intensities, since they together make up the basis for the mean intensities. Rather, the fact that it remained significant after adjustment for fitness, SED and MVPA separately indicates that these measures (number of prolonged bouts of SED and TPA) possess predictive validity for MetS. Some of these differences in results may be due to study design or differences in age of the participants

Looking at the different modalities one by one, cardiorespiratory fitness level was strongly related to prevalence of MetS in the present analyses. However, only the highest fitness-tertile displayed a significant lower prevalence compared to the lowest, reference tertile. This indicated that for middle-aged men and women in the present sample, fitness levels above 32 ml·min^-1^·kg^-1^ for women and 41 ml·min^-1^·kg^-1^ for men could be regarded as protective. It is important to remember that these analyses are based on tertiles, and these levels must be more precisely identified in a larger sample, with fitness analysed as a continuous variable. Previously, using other study designs and thresholds, values below 32 ml·min^-1^·kg^-1^ and 35 ml·min^-1^·kg^-1^ for women and men, respectively, have been reported in prospective studies as threshold values for higher risk of cardiovascular diseases [26]. These results, may contribute to form recommendations for desirable target-levels of fitness, in middle-aged populations.

The analyses on the relation between fitness, SED, LIPA, MVPA and the different sub-components of the MetS score (Figs 1–4), indicate differences in responses to the exposures, an observation that may be used to speculate on possible pathways involved. The effect of fitness is mainly associated with a lower waist circumference and fasting glucose levels, respectively. This is in line with findings from a Korean sample of 1008 middle aged participants [27]. In the above-referred analysis from the Cooper Center Longitudinal Study [14], fitness did not predict glucose nor waist circumference among the study participants, but a large proportion of the baseline participants were excluded from the analysis because of missing data, possibly leading to a biased selection. Although fitness level is mainly determined by the intensity and amount of recent exercise, it has a considerable genetic component (suggested to be 25 to 40%) [28]. Variation in fitness response to exercise as well as in intrinsic physiological factors such as oxidative capacity of the working muscle and individual variation in skeletal muscle fibre-types [29,30], may partly explain the independent association between fitness, MetS and its unfavourable sub-components.

Regarding MVPA, an association with unfavourable levels of all sub-components of the MetS was shown. Previous studies using self-reported MVPA as well as accelerometry have shown similar results [14,31]. However, this may well be the first study demonstrating this relationship, which remained strong, after simultaneous adjustment of objectively assessed SED and cardiorespiratory fitness level. This implies that MVPA seems to provide a general or possibly several specific changes in homeostasis to which the several systems need to respond and thereby make adaptions. Since MetS is made up by several sub-components, increased MVPA may be a first hand choice for lifestyle interventions.

Finally, the time spent in SED and its counter-form, LIPA, exhibited a strong relation to triglycerides, HDL-cholesterol and waist circumference, but not blood pressure. These findings are in line with previous epidemiological [16,32] as well as experimental studies [33]. These unfavourable responses are primarily counteracted by daily repeated periods of LIPA. One possible mechanism is the absence of muscular contraction during sitting, which will undermine the endocrine and paracrine functions of the skeletal muscle due to reduced release of so-called myokines [34]. Another is the adverse haemodynamic responses, such as low laminar shear stress, turbulent blood flow and blood pooling in the legs, which are induced by even short periods of sitting (30 to 60 min) [35]. Importantly, SED is not totally independent of LIPA but show a rather strong inverse relationship, which may also contribute to the effect of respective PA-pattern modalities.

Our second aim of the study was to assess the efficacy of two different accelerometry analyses. Importantly, triaxial accelerometry seemed more predictive compared to uniaxial accelerometry for the detection of MetS, based on the fact that uniaxial accelerometry yielded ORs closer to 1 and fewer significant ORs, compared to triaxial assessment. The differences between modes of accelerometry were most pronounced in the lower intensities, which is partly contradictory to a previous validation study in the laboratory setting [36], where uni- and triaxial Actigraph accelerometry registrations predicted low intensity PA, to a similar degree. As a result of our study, using the triaxial vector magnitude in future analysis or clinical practice when evaluating the daily PA pattern, and mainly at the lower end of the intensity spectrum, thus seems highly justified. The different predictive validity of uni- and tri-axial accelerometry may well be placement dependent. In the present study, hip-worn accelerometers were used. Any differences in validity between analyse modes regarding for example wrist-worn or chest-mounted remain to be investigated. Further, the use of combining monitors (multiple accelerometers or accelerometers plus inclinometers) or different machine learning procedures may provide even more valid measures of activity pattern and possibly also higher predictive validity.

In addition to the tertile evaluation of MVPA and its relation to MetS, we performed an extended analysis to investigate the importance of fulfilment of two different proposed PA recommendations (strict or less strict, as described above), in which both recommendations yielded similar reduction of OR compared to participants not fulfilling the recommendations. This implies that fulfilment of current PA recommendations, using accelerometry, seems to be independent of how MVPA is accumulated during the week. The non-significant OR reported in the results section for the current PA recommendations may partly be dependent on the fact that fewer individuals were classified as physically active using these recommendations, resulting in limited power. However, it is essential to acknowledge that the more objective PA assessment method of accelerometry shows entirely different MVPA values, compared to self-assessed PA questionnaires. As the current PA recommendations are bases on self-assessed PA levels, this must be taken into consideration when clinically implementing the above results.

Finally, a recently highlighted research area is the findings of prolonged sitting to be an independent important for health and longevity regardless of MVPA [37]. These studies have most often used regression-based analysis (similar to the present ones), including MVPA as an independent variable when studying the association between SED and the outcome of interest. However, the question remains if avoiding prolonged sitting is equally important for those performing different amounts of MVPA. Therefore, we stratified MVPA into low (below median) and high (above median) and studied the importance of increased SED tertiles for MetS prevalence. As higher tertiles of SED increased the risk of MetS (2.5-fold for second tertile and 3.5-fold for third tertile) in participants with low MVPA, high level of MVPA seemed to attenuate the risk of SED. Participants with high MVPA but in the highest tertile of SED, showed a more than 2-fold increased risk of MetS, however only borderline significant. This is in line with previous results on the association between self-reported SED, MVPA and all-cause mortality death rates, which reported a strong association with increased SED in inactive participants, but also an increased death-rate for active individuals with the highest SED [38]. However, the present study adds to the current knowledge, by showing that otherwise more active individuals, may be partly, but not entirely, protected from the negative effects of being sedentary.

### Clinical implications

The present results have several important clinical implications. Firstly, the finding that all aspects of the daily PA pattern (SED, LIPA and MVPA) were independently, adjusted for each other and fitness, associated to MetS prevalence, is important in the clinical setting. Thus, all these aspects of the individual PA patterns must be taken into consideration. Hence, not only “exercise” or MVPA should be evaluated and prescribed (as generally recommended today), but it is also important to recommend limited daily time spent sitting. Substituting sedentary time by increasing LIPA should be a target for behaviour change, not least in inactive individuals at high risk. Secondly, cardiorespiratory fitness remains the strongest predictor of MetS in the present analyses, and evaluation of fitness level, through maximal or submaximal testing, should be encouraged in clinical practice. The current trend of reduced use of the exercise test in the clinical setting is very unfortunate, also because of its usefulness for helping the clinician to prescribe the proper intensity level of activity to patients. The results of the present study, together with the increasing awareness of PA as an effective treatment modality in health care, could help to put focus on this issue. Thirdly, reduction of SED is especially important in otherwise inactive individuals (low MVPA), as MVPA seems to partly “protect” inactive individuals with respect to the risk of developing MetS.

Fourthly, fulfilment of current PA recommendations, using accelerometry, seems to be independent of how the MVPA is accumulated during the week. As most PA recommendations are based on self-assessed PA levels, these findings must be interpreted cautiously, however. Finally, the present study also shows that the triaxial accelerometry may be more useful in the clinical setting, being more sensitive in the lower end of the PA spectrum, where many individuals are residing. The use of triaxial accelerometry is thus indicated in the evaluation of PA interventions in the clinical setting of older, inactive persons.

How to form attractive and effective interventions to reduce extended time spent in sedentary behaviour and increase total volume of body movement is still only partly known. For instance, persons may be physically inactive due to age and/or disease, making the potential use of SED as predictor and outcome measure for PA interventions particularly useful in this population.

### Strengths and limitations

This is the first study to observe relations between MetS and a full panel of objectively assessed PA pattern as well as cardiorespiratory fitness. However, the cross-sectional design of the present study limits the possibility to study causality of the observed associations. The method used for estimating aerobic fitness in this study did not use direct measures of gas exchange at maximal rates of work. This introduces a limitation, as all indirect estimates introduce some measurement error. Further, a total of 130 participants did not perform the fitness test, some of them due to on-going illness or in some cases body mass exceeding the maximal user weight for the ergometer. This exclusion may have affected the results to some degree; possibly by an underestimation of the association between MetS and fitness. Another possible limitation lies in the fact that MetS is defined in several different manners and the choice to use the NCEP ATP-III form can be seen as arbitrary. Using the regression analyses technique when controlling for interrelations is one of several possible techniques. The issue of multi-collinearity, where independent variables correlate strongly (r>0.7) with each other limits the analyses. These study regressions were used for comparability to other publications.

### Conclusion

All aspects of the individual PA-pattern and fitness measurements are independently associated to the MetS. While fitness remains the strongest predictor of the MetS, this study for the first time, show that the associations remain, when simultaneously adjusting for both SED time and MVPA. Triaxial accelerometry seems more sensitive in the lower range of the PA spectrum, thereby being more clinically useful. Time spent sedentary is especially important to consider in patients with low activity elsewhere (low MVPA). The results of this study also reinforce the clinical use of PA recommendations, not least in patients at risk of MetS.
