# Correction: Cardiorespiratory Fitness, Sedentary Behaviour and Physical Activity Are Independently Associated with the Metabolic Syndrome, Results from the SCAPIS Pilot Study

**DOI:** 10.1371/journal.pone.0197801

**Published:** 2018-05-22

**Authors:** Örjan Ekblom, Elin Ekblom-Bak, Annika Rosengren, Mattias Hallsten, Göran Bergström, Mats Börjesson

There are errors in Figs 1–5. There are also errors in Table 3 and Table 4. The corrected figures and tables are based on the NCEP Adult Treatment Panel III (ATPIII panel). Please see the correct Figs 1–5 and the correct Table 3 and Table 4 below.

**Fig 1:**
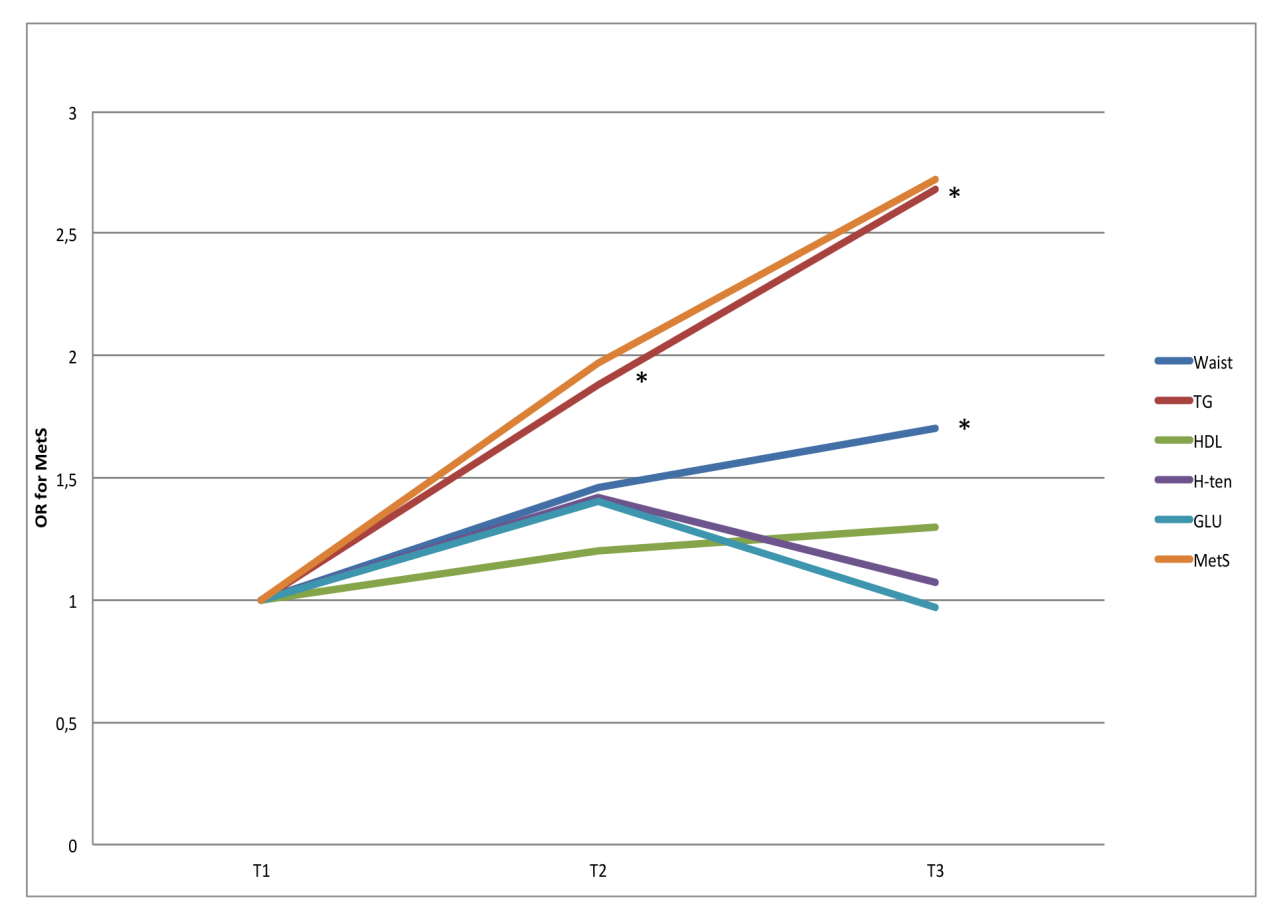
OR for MetS for tertiles of sedentary time. ORs are adjusted for gender, age, education level (university degree vs. not), energy intake (kcal·d^-1^ in quartiles), smoking habits (regular smoker vs. not) and psycho-social stress (self-reported into four levels), estimated VO_2_max (ml·min^-1^·kg^-1^, in tertiles) and % of wear time spent in MVPA (in tertiles). * Denotes significant difference to reference group.

**Fig 2:**
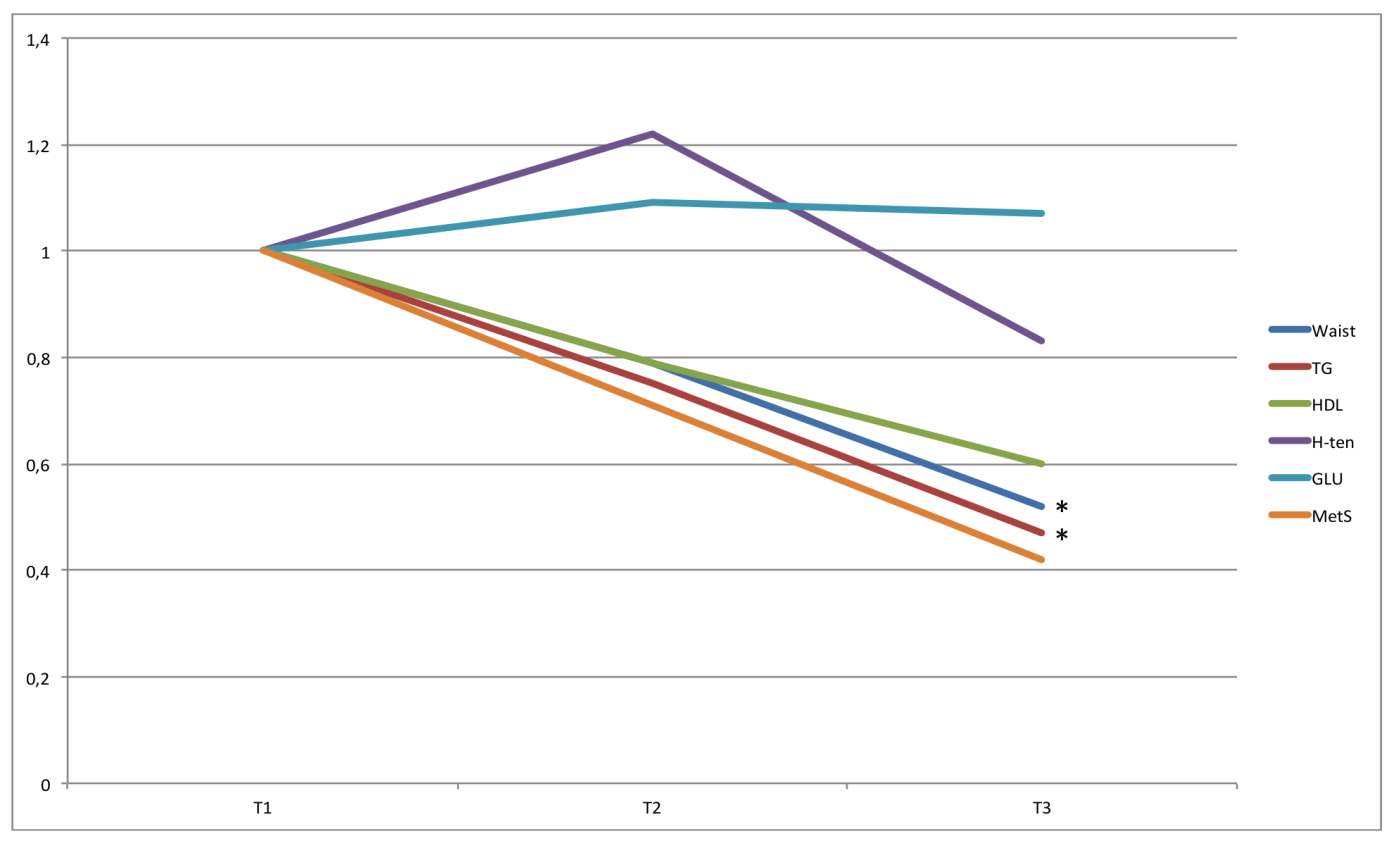
OR for MetS for tertiles of time in LIPA. ORs are adjusted for gender, age, education level (university degree vs. not), energy intake (kcal·d^-1^ in quartiles), smoking habits (regular smoker vs. not) and psycho-social stress (self-reported into four levels), % of wear time spent in MVPA (in tertiles), and estimated VO_2_max (ml·min^-1^·kg^-1^, in tertiles). * Denotes significant difference to reference group.

**Fig 3:**
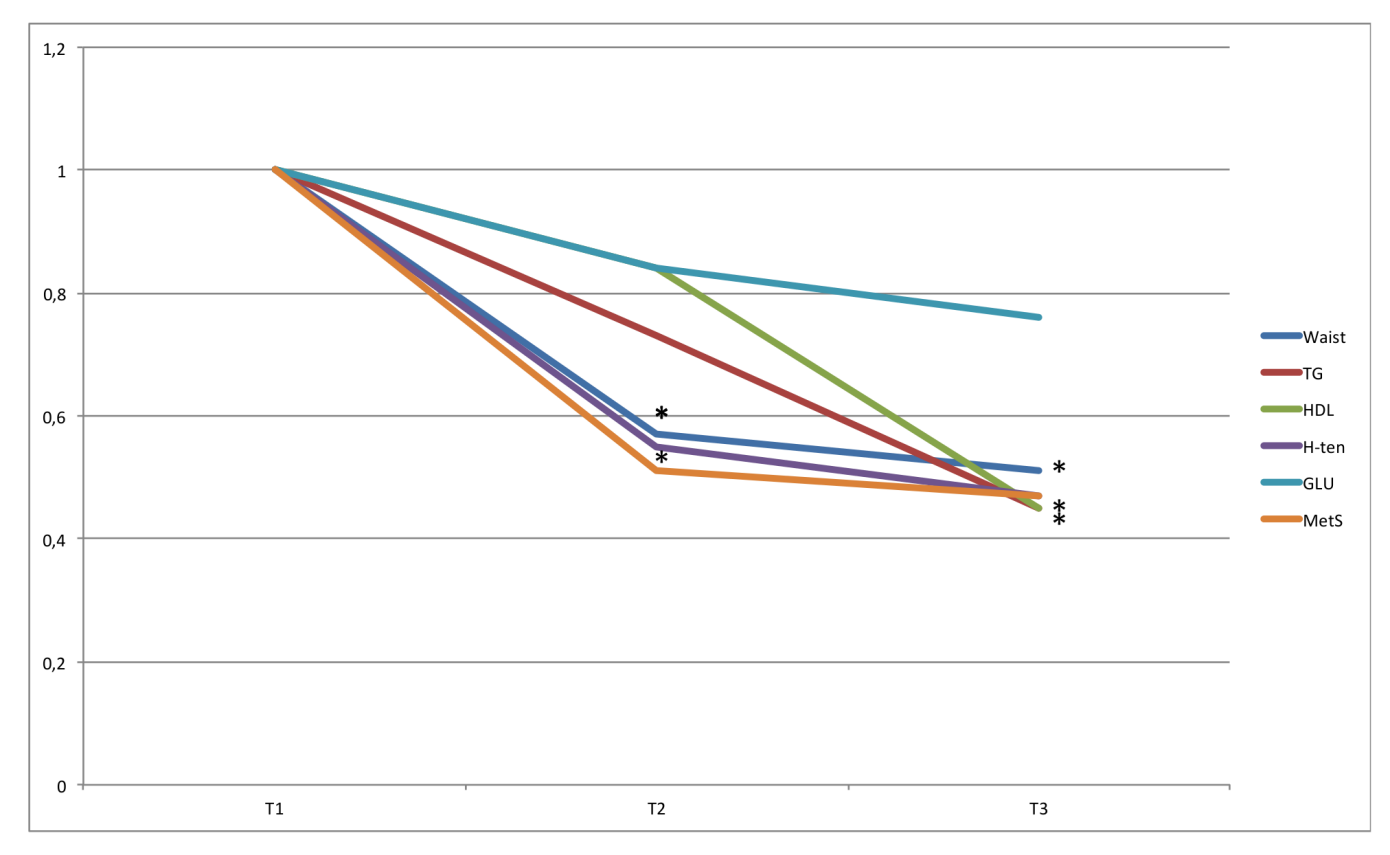
OR for MetS for tertiles of time in MVPA. ORs are adjusted for gender, age, education level (university degree vs. not), energy intake (kcal·d^-1^ in quartiles), smoking habits (regular smoker vs. not) and psycho-social stress (self-reported into four levels), % of wear time spent in SED (in tertiles), and estimated VO_2_max (ml·min^-1^·kg^-1^, in tertiles). * Denotes significant difference to reference group.

**Fig 4:**
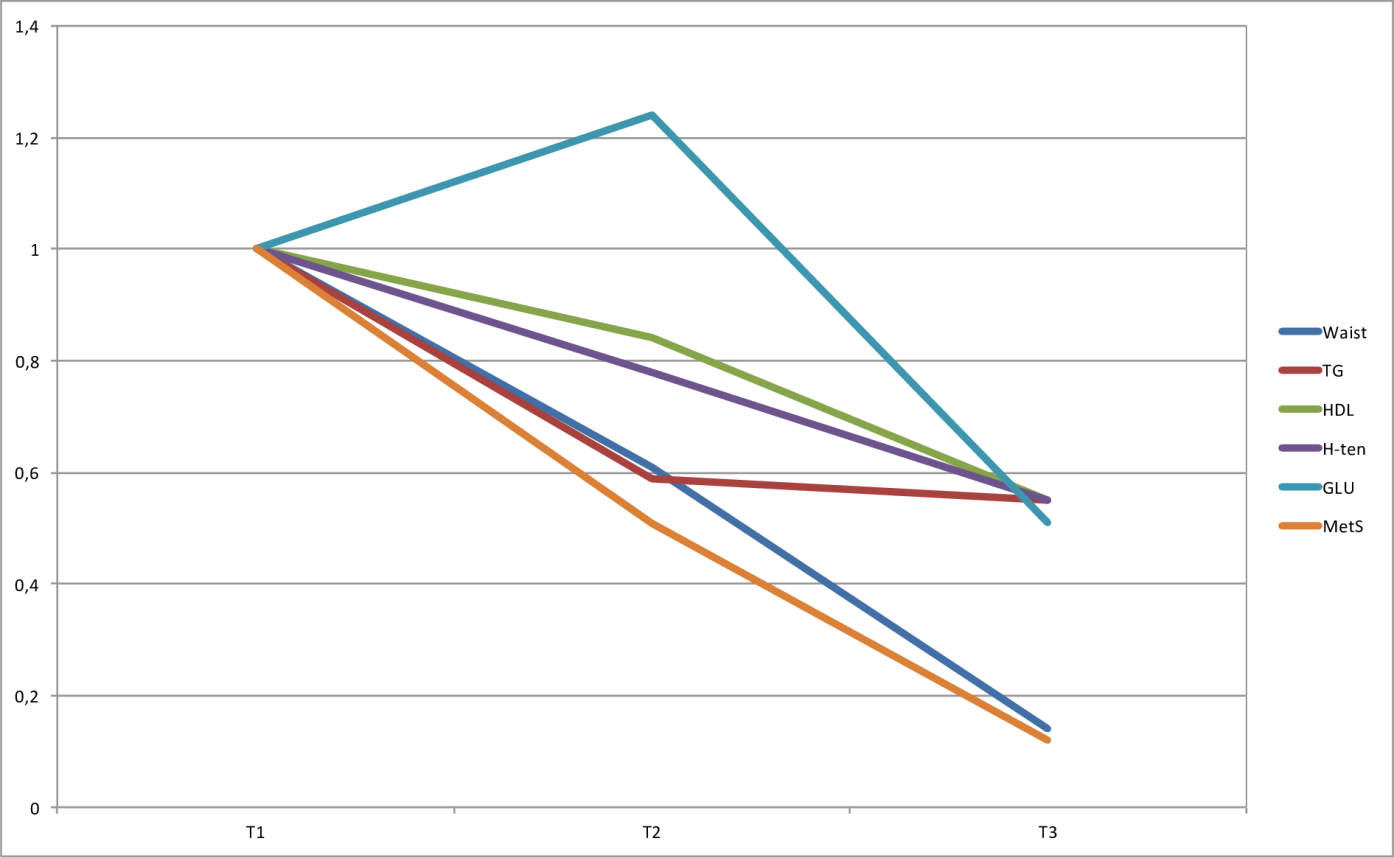
OR for MetS for tertiles of fitness. ORs are adjusted for gender, age, education level (university degree vs. not), energy intake (kcal·d^-1^ in quartiles), smoking habits (regular smoker vs. not) and psycho-social stress (self-reported into four levels), % of wear time spent in SED (in tertiles), and % of wear time spent in MVPA (in tertiles). * Denotes significant difference to reference group.

**Fig 5:**
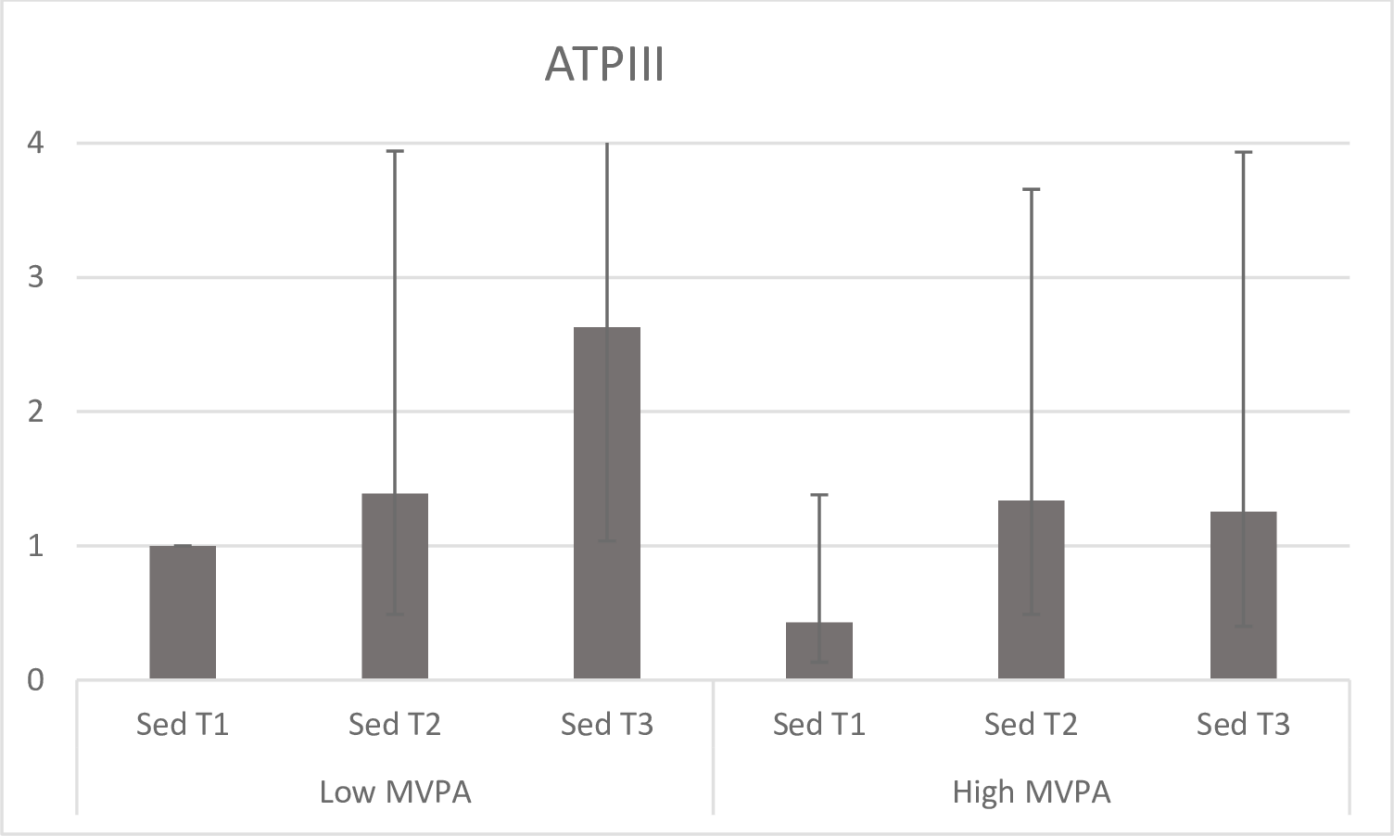
Stratified analysis across SED tertiles in high (above median) and low (below median) MVPA. ORs are adjusted for gender, age (yrs.), education level (university degree vs. not), energy intake (kcal·d^-1^, in quartiles), smoking habits (regular vs. not), psychosocial stress (self-reported into four levels) and fitness (in tertiles).

**Table 3:** Analyzed using ATPIII-cutoffs for uniaxial accelerometry.

			Tertile 2	Tertile 3
**Fitness**		n		
	Age-gender	784	**0.50 (0.31–0.74)**	**0.09 (0.04–0.18)**
	+Lifestyle	709	**0.41 (0.26–0.67)**	**0.08 (0.04–0.18)**
	+SED	630	**0.46 (0.27–0.79**)	**0.11 (0.05–0.29)**
	+MVPA	630	**0.50 (0.29–0.86)**	**0.12 (0.05–0.28)**
	+SED and MVPA	630	**0.49 (0.29–0.85)**	**0.12 (0.05–0.29)**
**Accelerometry**				
**SED**	Age-gender	932	1.32 (0.85–2.07)	**1.83 (1.19–2.81)**
	+Lifestyle	831	1.59 (0.97–2.61)	**2.37 (1.47–3.83)**
	+MVPA	831	1.37 (0.82–2.26)	**1.80 (1.09–2.96)**
	+Fitness	630	1.74 (0.89–3.42)	**2.45 (1.26–4.79)**
	+MVPA and Fitness	630	1.55 (0.78–3.10)	1.90 (0.95–3.89)
**LIPA**	Age-gender	932	0.85 (0.57–1.27)	0.72 (0.47–1.11)
	+Lifestyle	831	0.76 (0.49–1.16)	**0.56 (0.35–0.90)**
	+MVPA	831	0.81 (0.53–1.26)	**0.61 (0.38–0.98)**
	+Fitness	630	0.92 (0.53–1.61)	0.52 (0.27–1.01)
	+MVPA and Fitness	630	0.97 (0.55–1.71)	0.52 (0.26–1.02)
**MVPA**	Age-gender	932	**0.33 (0.21–0.50)**	**0.31 (0.20–0.47)**
	+Lifestyle	831	**0.34 (0.22–0.54)**	**0.33 (0.21–0.52)**
	+SED	831	**0.36 (0.23–0.57)**	**0.39 (0.24–0.63)**
	+Fitness	630	**0.26 (0.14–0.50)**	**0.31 (0.17–0.57)**
	+SED and Fitness	630	**0.28 (0.14–0.53)**	**0.36 (0.19–0.67)**
**TPA**	Age-gender	932	**0.36 (0.23–0.54)**	**0.35 (0.23–0.54)**
	+Lifestyle	831	**0.34 (0.21–0.53)**	**0.32 (0.22–0.55)**
	+SED	831	**0.37 (0.22–0.60)**	**0.40 (0.23–0.71)**
	+MVPA	831	**0.46 (0.27–0.77)**	0.68 (0.32–1.44)
	+Fitness	630	**0.42 (0.23–0.76)**	**0.33 (0.17–0.62)**
	+SED, MVPA and Fitness	630	0.84 (0.38–1.84)	1.31 (0.39–4.46)
**SED bouts**	Age-gender	930	1.07 (0.68–1.70)	**1.73 (1.41–2.64)**
	+Lifestyle	831	1.20 (0.74–1.95)	**2.21 (1.40–3.49)**
	+MVPA	831	1.09 (0.67–1.80)	**1.81 (1.13–2.90)**
	+Fitness	630	0.96 (0.50–1.86)	**1.97 (1.06–3.64)**
	+MVPA and Fitness	630	0.92 (0.47–1.81)	1.67 (0.89–3.15)
**SED breaks**	Age-gender	932	0.74 (0.50–1.11)	**0.61 (0.40–0.93)**
	+Lifestyle	831	**0.63 (0.41–0.98)**	**0.50 (0.31–0.80)**
	+MVPA	831	0.69 (0.44–1.07)	**0.56 (0.35–0.91)**
	+Fitness	630	0.61 (0.34–1.09)	0.54 (0.28–1.03)
	+MVPA and Fitness	630	0.68 (0.38–1.23)	0.56 (0.29–1.09)

**Table 4:** Analyzed using ATPIII-cutoffs for triaxial accelerometry.

			Tertile 2	Tertile 3
**Fitness**		n		
	Age-gender	784	**0.50 (0.31–0.74)**	**0.09 (0.04–0.18)**
	+Lifestyle	709	**0.41 (0.26–0.67)**	**0.08 (0.04–0.18)**
	+SED	633	**0.51 (0.30–0.86)**	**0.11 (0.05–0.27)**
	+MVPA	633	**0.49 (0.29–0.83)**	**0.12 (0.05–0.27)**
	+SED and MVPA	633	**0.51 (0.30–0.88)**	**0.12 (0.05–0.28)**
**Accelerometry**				
**SED**	Age-gender	938	1.28 (0.80–2.03)	**2.47 (1.60–3.83)**
	+Lifestyle	835	1.40 (0.83–2.35)	**3.15 (1.95–5.10)**
	+MVPA	835	1.24 (0.73–2.11)	**2.45 (1.46–4.12)**
	+Fitness	633	2.23 (1.09–4.58)	**3.53 (1.76–7.09)**
	+MVPA and Fitness	633	1.97 (0.95–4.11)	**2.72 (1.31–5.63)**
**LIPA**	Age-gender	938	**0.56 (0.37–0.83)**	**0.50 (0.33–0.77)**
	+Lifestyle	835	**0.49 (0.32–0.75)**	**0.37 (0.23–0.59)**
	+MVPA	835	**0.54 (0.35–0.84)**	**0.42 (0.26–0.68)**
	+Fitness	633	0.63 (0.36–1.10)	**0.40 (0.21–0.77)**
	+MVPA and Fitness	633	0.71 (0.40–1.26)	**0.42 (0.22–0.81)**
**MVPA**	Age-gender	938	**0.43 (0.29–0.65)**	**0.39 (0.26–0.59)**
	+Lifestyle	835	**0.44 (0.28–0.69)**	**0.32 (0.24–0.60)**
	+SED	835	**0.52 (0.33–0.83)**	**0.55 (0.33–0.90)**
	+Fitness	633	**0.43 (0.24–0.79)**	**0.36 (0.19–0.66)**
	+SED and Fitness	633	**0.51 (0.28–0.95)**	**0.47 (0.24–0.90)**
**TPA**	Age-gender	930	**0.36 (0.25–0.55)**	**0.35 (0.23–0.54)**
	+Lifestyle	835	**0.56 (0.36–0.86)**	**0.33 (0.20–0.53)**
	+SED	835	0.76 (0.46–1.24)	0.58 (0.30–1.12)
	+MVPA	835	0.65 (0.40–1.08)	**0.44 (0.22–0.90)**
	+Fitness	633	0.58 (0.33–1.02)	**0.32 (0.16–0.62)**
	+SED, MVPA and Fitness	633	1.31 (0.57–2.99)	1.42 (0.39–5.21)
**SED bouts**	Age-gender	930	1.06 (0.68–1.66)	**1.73 (1.14–2.63)**
	+Lifestyle	835	1.16 (0.71–1.89)	**2.24 (1.42–3.53)**
	+MVPA	835	1.08 (0.66–1.76)	**1.79 (1.12–2.87)**
	+Fitness	633	1.35 (0.70–2.58)	**2.30 (1.22–4.32)**
	+MVPA and Fitness	633	1.27 (0.66–2.49)	1.80 (0.93–3.48)
**SED breaks**	Age-gender	938	0.74 (0.50–1.10)	**0.52 (0.34–0.81)**
	+Lifestyle	835	0.66 (0.43–1.02)	**0.47 (0.29–0.75)**
	+MVPA	835	0.76 (0.49–1.17)	**0.55 (0.34–0.88)**
	+Fitness	633	0.85 (0.48–1.52)	0.54 (0.29–1.02)
	+MVPA and Fitness	633	1.06 (0.58–1.92)	0.65 (0.34–1.24)
